# Optical determination of Shockley-Read-Hall and interface recombination currents in hybrid perovskites

**DOI:** 10.1038/srep44629

**Published:** 2017-03-20

**Authors:** Valerio Sarritzu, Nicola Sestu, Daniela Marongiu, Xueqing Chang, Sofia Masi, Aurora Rizzo, Silvia Colella, Francesco Quochi, Michele Saba, Andrea Mura, Giovanni Bongiovanni

**Affiliations:** 1 Dipartimento di Fisica, Università degli Studi di Cagliari, I-09042 Monserrato, Italy; 2 Istituto di Nanotecnologia CNR-Nanotec, Polo di Nanotecnologia c/o Campus Ecotekne, via Monteroni, 73100 Lecce, Italy; 3 Dipartimento di Matematica e Fisica “E. De Giorgi”, Università del Salento, Via Arnesano snc, 73100 Lecce, Italy

## Abstract

Metal-halide perovskite solar cells rival the best inorganic solar cells in power conversion efficiency, providing the outlook for efficient, cheap devices. In order for the technology to mature and approach the ideal Shockley-Queissier efficiency, experimental tools are needed to diagnose what processes limit performances, beyond simply measuring electrical characteristics often affected by parasitic effects and difficult to interpret. Here we study the microscopic origin of recombination currents causing photoconversion losses with an all-optical technique, measuring the electron-hole free energy as a function of the exciting light intensity. Our method allows assessing the ideality factor and breaks down the electron-hole recombination current into bulk defect and interface contributions, providing an estimate of the limit photoconversion efficiency, without any real charge current flowing through the device. We identify Shockley-Read-Hall recombination as the main decay process in insulated perovskite layers and quantify the additional performance degradation due to interface recombination in heterojunctions.

Six decades after the demonstration of the first silicon photovoltaic cell^1^, only a limited number of semiconductors enable single junction photovoltaic devices with power conversion efficiencies (PCEs) exceeding 20%^2^. Among these, hybrid organic-inorganic perovskites (HPs) represent an emerging class of solution-processed semiconductors^3^,^4^,^5^,^6^,^7^,^8^,^9^,^10^ with the potential to approach the Shockley-Queisser limit of the PCE^11^ and the prospect to be integrated with established commercial technologies to fabricate cheap, multi-junction solar cells^12^,^13^, with even higher efficiencies.

The rapid increase in photovoltaic performance has been accompanied by intense research on the photophysics of these materials^14^,^15^,^16^,^17^,^18^,^19^,^20^,^21^,^22^. A key aspect of photoconversion in HPs is the recombination current, a ubiquitous mechanism of energy loss in solar cells. Photovoltaic devices operate as non-ideal current generators in which a fraction of photogenerated electrons and holes recombines inside the cell, feeding the internal diode current instead of being injected into the external load^23^. Minimal recombination energy losses are achieved in the ideal case in which electron-hole pairs decay only radiatively; in this regime, the recombination resistance is maximized, and so is the cell voltage. The external electroluminescence quantum yield (*EQY*_EL_) quantifies the amount of radiative recombination with respect to non-radiative losses; the best solar cells show the highest *EQY*_EL_ (10^−3^–10^−1^) and therefore operate at a voltage as close as possible to the semiconductor band gap—up to 0.78 *E*_*g*_ in GaAs^24^ and very recently 0.76 *E*_*g*_ in HP^8^,^20^,^25^,^26^,^27^.

The simplest architecture of heterojunction solar cells consists of a light absorber sandwiched between two charge-selective semiconductor layers, one of which allows the flow of photoexcited electrons (ETL) but blocks the transmission of holes, while the other conveys only photoexcited holes (HTL) to the opposite electrode (Fig. 1). Nonradiative decay channels are typically associated with two possible mechanisms: one is related to the presence of intragap recombination centres in the absorber; the other one is due to the recombination at the two heterojunctions between the absorber and the transport layers (HTL or ETL), such as back-recombination of electrons (holes) collected by the ETL (HTL) with holes (electrons) accumulating at the interface with the absorber. Information on which annihilation process dominates can be inferred from the ideality factor *m* of the diode current^28^,^29^, typically extracted by fitting the charge current-voltage (*I*-*V*) characteristics. However, given the logarithmic dependence of the cell voltage on the recombination rate, the determination of the ideality factor is not very reliable, especially for HP solar cells, where hysteresis and degradation effects may lead to distortion of the I-V characteristics. So far, the values reported for HPs mainly lie in the range 1.7–2^30^,^31^,^32^,^33^,^34^, but extend as far down as 1.2 and up to 5^35^,^36^. As a consequence, it is difficult to identify from the electrical characterization what recombination processes limit the photoconversion efficiency, and therefore to elaborate an informed strategy to improve the devices.

Here we propose an optical experimental method to estimate the voltage drop caused by each type of nonradiative channel. Instead of the *I*-*V* curves, we studied the free energy of the electron-hole pairs (*μ*) as a function of the intensity of the exciting light (*I*_ex_), namely the *μ*-*I*_ex_ characteristics. We then show how to get information on the ideality factor and recombination channels. Since no charge current flows, the optical approach can be used to study the double heterojunctions composing the solar cells, as well as the two single heterojunctions and the absorbing layer alone, thereby providing a comparative method to discriminate between interface and bulk recombination.

## Results

### All-optical determination of the electron-hole free energy

According to the theory of non-equilibrium semiconductors, the free energy (or chemical energy) of photogenerated electron-hole pairs, *μ*, is equal to the energy splitting of the quasi-Fermi levels of electrons in the conduction (*F*_cb_) and valence (*F*_vb_) bands, respectively. To provide an expression for *μ* in terms of measurable optical quantities, we consider Kirchhoff’s law of radiation, which represents the detailed balance between emission and absorption, generalized by Würfel to account for non-equilibrium electron and hole populations^37^:


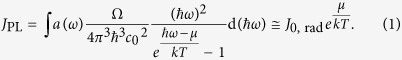


**Figure 1:**
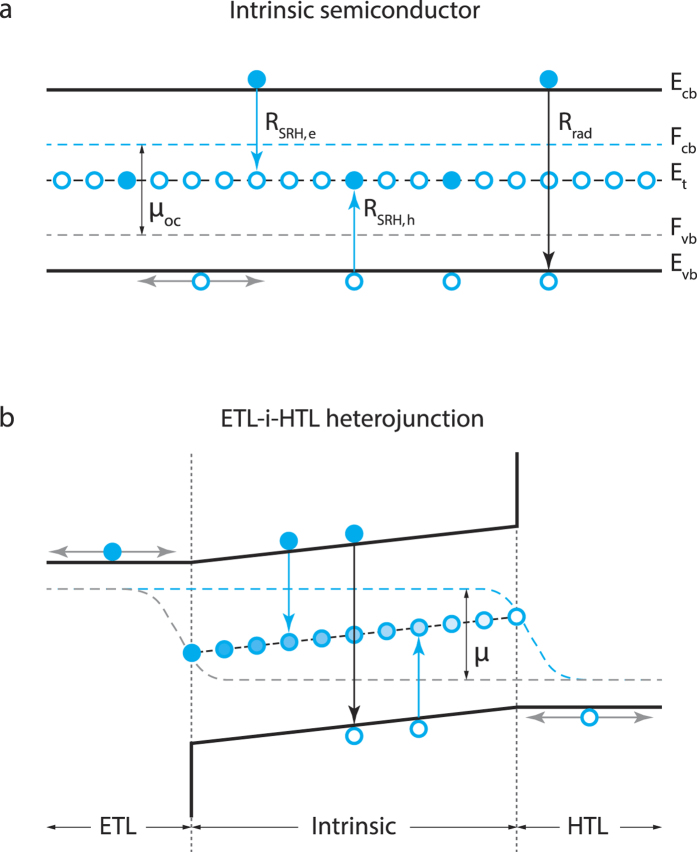
Electron and hole energetics and recombinations in perovskites and perovskite-based solar cells. (**a**) Stand-alone intrinsic layer. The free energy *μ*_oc_ of photogenerated electron-hole pairs is equal to the energy splitting of the quasi-Fermi levels of electrons in the conduction (*F*_cb_) and valence (*F*_vb_) bands. No electric voltage is present between the two sides due to the absence of electron- and hole-selective contacts. An empty trap level *E*_*t*_ in the mid-gap is assumed. *R*_SRH,*e(h*)_ is the Shockley-Read-Hall recombination rate of electrons (holes) per unit of volume. Correspondingly, *R*_rad_ refers to radiative recombinations. (**b**) HTL-i-ETL double heterojunction. The difference between *F*_cb_ in the ETL and *F*_vb_ in the HTL is given by *μ* = *eV*, where *V* is the circuit voltage of the solar cell in absence of electrical losses. As shown in Supplementary Fig. 2, equilibrium conditions of the solar cell in the dark impose that the concentration of trapped electrons varies across the intrinsic layer: trap levels at the centre of the i-semiconductor traps are half-filled so that the recombination rate *R*_SRH,*e(h*)_ of the excess carriers is quite large. Conversely, trap levels close to the ETL (HTL) are filled (empty), no trapping of electrons (holes) by mid-gap states is thereby possible, leading to negligible *R*_SRH,*e(h*)_ values.

*J*_*PL*_ is the emitted photon current density, proportional to the external photoluminescence intensity, while *a(ω*) denotes the absorptivity, which depends both on the absorption coefficient and film thickness. Ω is the effective external emission angle. The right-hand side of equation (1) is valid for excitation levels typical of solar illumination, for which the Bose function can be substituted by the Boltzmann distribution. Equation (1) provides the analytical relation between *J*_PL_, *μ*, and the photon current density *J*_0,rad_ emitted by the semiconductor in thermal equilibrium with the environment (*μ* = 0)^38^. To our purposes, *J*_PL_ can be opportunely expressed in terms of the external photoluminescence quantum yield (*EQY*_PL_), defined as the ratio between *J*_*PL*_ and the absorbed excitation photon flux, that is 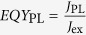. With this substitution, equation (1) can be reformulated to explicitly link *μ* to *EQY*_PL_:


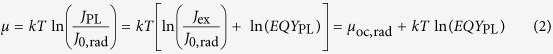


in which 
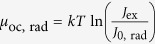
 represents the upper limit for the free energy when only radiative decays occur (*EQY*_PL_ = 1) and in the open circuit condition. If all incident photons with energy higher than the optical gap are absorbed, 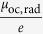 represents the Shockley-Queisser limit to the open circuit voltage *V*_oc_^7^. The second term of the right-hand side of equation (2) states a simple but very useful rule: nonradiative decay channels affect the free energy only through the *EQY*_PL_, leading to a free-energy loss of In(10^−1^) *kT* = 60 meV for a factor of 10 drop of *EQY*_PL_ at 300 K. Furthermore, equation (2) provides a reformulation of Kirchhoff’s law that allows us to highlight similarities between the optical and the optoelectronic reciprocity relations^39^. The latter ones, proposed ten years ago by Rau, connect the electroluminescent emission and photovoltaic voltage through the equations *eV*_oc_ = *eV*_oc,rad_ + *kT* ln(*EQY*_EL_) and 
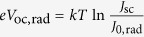
, where *e*_*J*0,rad_ represents the radiative charge current in the dark, and *eJ*_sc_ the short-circuit charge current (note that here the symbol *J* is used for particle currents, not charge currents, hence the need to multiply currents by the electron charge *e*). Figure 1 visualizes *μ* for a stand-alone intrinsic semiconductor layer and for a double heterojunction (HTL-i-ETL; ‘i’ stands for the intrinsic absorber semiconductor), providing the link between *μ* and the circuit voltage in solar cells. In the stand-alone layer (Fig. 1a), *μ* = *μ*_*oc*_ is constant throughout the film but no external electric voltage is produced due to the absence of electron- and hole-selective contacts. In the HTL-i-ETL solar cell (Fig. 1b), the free energy is provided by the difference between the energies of the quasi-Fermi levels of electrons in the ETL and in the HTL at the opposite side of the device. 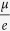 in the intrinsic layer equates the circuit voltage *V* of the solar cell if *F*_cb_ and *F*_vb_ do not vary from the transport materials up to the external contacts.

### All-optical determination of the ideality factor

The optical reciprocity relation in eq. (2) allows measuring *μ* without having any information on the specific microscopic mechanisms of electron-hole recombination. Nonetheless, as we are interested in uncovering the decay channels, we need to find out how these processes influence the *μ*-*J*_ex_ characteristics. We therefore need to specify a microscopic model for recombinations. Let us consider the simplest case, in which decays of electrons and holes are driven by elementary processes such as monomolecular, bimolecular, or higher order multi-particle interactions. If the corresponding recombination rates for electrons and holes follow a power law 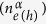, the relation 
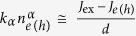
 holds under steady-state excitation (neglecting thermal excitation/recombination processes)^40^, where 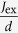 represents the mean carrier generation rate per unit of volume, 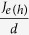 the electron (hole) extraction rate per unit of volume, and *α* denotes the number of carriers involved in the recombination process (*α* = 1,2,3 for monomolecular, bimolecular, and trimolecular recombinations respectively). To connect *μ* to the free electron (*n*_*e*_) and hole (*n*_*h*_) concentrations, we use the mass action law, generalized to account for non-equilibrium carriers:


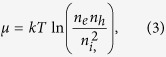


where *n*_*i*_ is the free-carrier concentration of the intrinsic semiconductor in the dark. Defining 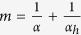, eq. (3) can be rewritten as:


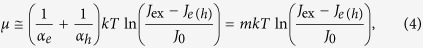


in which *J*_0_ is a constant. For optoelectronic measurements (*J*_*e(h*)_ ≠ 0), eq. (4) is often written by replacing *μ* = *eV* in the form 
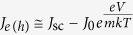
, where *m* is the ideality factor of the *μ*-*J*_ex_ curves, with *J*_0_ being the reverse bias saturation current (note again that charge currents are usually considered instead of particle currents as we are doing here). When *J*_*e(h*)_ = 0 (*μ* = *μ*_oc_), eq. (4) provides an all-optical route to determine the ideality factor *m* and consequently to identify the recombination mechanisms. Elementary electron-hole annihilation processes (*α* = 1,2,3,…) are associated with rational values of *m*. Band-to-band electron-hole recombinations (*n*_*e*_~*n*_*h*_,*α*_*e*_ = *α*_*h*_ = 2,) yield *m* = 1; nonradiative monomolecular decays of minority carriers in doped semiconductors 
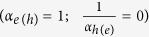
 also lead to *m* = 1. Auger recombinations are trimolecular processes, so 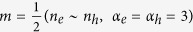
28. Shockley-Read-Hall (SRH) nonradiative decays in the space-charge region of a p-n junction or in the intrinsic layer of a p-i-n (HTL-i-ETL) device, for which *α*_*e*_ = *α*_*h*_ ≅ 1, is instead characterized by *m* = 2 (see Supplementary Note 1)^29^.

### Free energy and ideality factor in perovskite films

**Figure 2:**
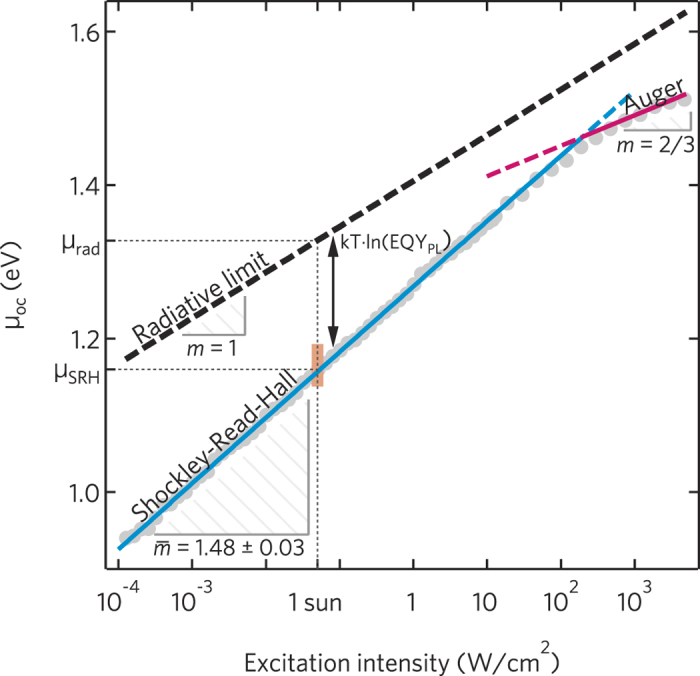
*μ* -*I*_ex_ characteristics of intrinsic MAPbI_3_ films. Grey markers represent the measured free energy *μ*_oc_ as a function of the excitation intensity (*I*_ex_) delivered by a CW laser at 532 nm. The orange box encloses the dispersion of *μ*_oc_ for samples fabricated with a variety of different techniques. For 1 mW/cm^2^ < *I*_ex_ < 100 W/cm^2^, *μ*_oc_ depends linearly on ln(*I*_ex_) with a slope *m* of approximately 3/2 regardless of the fabrication method, yielding an average ideality factor 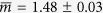 (within one standard deviation). Shockley-Read-Hall recombinations are expected to lead to a rational ideality factor *m* = 3/2 at low illumination levels. The experimental slope decreases to approximately 2/3 for *I*_ex_ > 100 W/cm^2^, as foreseen for Auger recombination. At *I*_ex_ = 50 mW/cm^2^, the rate of photons absorbed by the film matches that one obtained at an illumination level of one sun (AM 1.5G). At this excitation intensity, *μ*_rad_ = 1.33 eV while 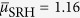 eV, with a free energy loss due to Shockley-Read-Hall recombinations 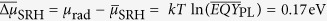.

The experimental data from single HP layers allow to understand the bulk carrier recombination processes and to determine the maximum attainable free energy *μ*_oc_. Figure 2a reports *μ*_oc_ as a function of ln(*I*_ex_), where *I*_ex_ is the excitation light intensity delivered by a green CW laser (*λ* = 532 nm). *μ*_oc_ was determined from the *EQY*_PL_ of MAPbI_3_ as a function of *I*_ex_. We analysed perovskite layers grown with several deposition methods, namely single-step and double-step techniques. According to eq. (2), it is sufficient to assess *μ*_oc,rad_ at just one excitation intensity, which we chose so that the absorbed photon current density of the green light and solar light at AM1.5G (*J*_sun_, *ħω* > *E*_OG_) were equal (*I*_ex_ = 50 mW/cm^2^). In the Supplementary Note 2, we show that *μ*_oc,rad_ can be usefully written as:


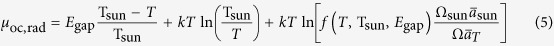


In this equation, *E*_gap_ = 1.602 eV is the photon energy of the optical gap (see Supplementary Note 2); 
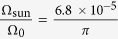
 takes into account the fact that the sun illuminates only a portion of the effective solid angle seen by the semiconductor^37^; 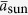 and 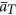 represent the spectrally averaged film absorptivity, weighted by the black-body radiation spectra at T_sun_ = 5541 K and *T* = 300 ± 20 K, respectively. The temperature *T* of the electron-hole gas was determined by fitting a Boltzmann distribution to the high-energy tail of the photoluminescence spectrum. *f(T*, T_sun_, *E*_gap_) is an analytical function, whose value can be readily calculated as equal to 1.718. In the Shockley-Queisser limit, 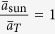, hence the radiative free energy only depends on T_sun_ and properties of the material, namely *E*_gap_ and the effective working temperature *T*. We then obtained *μ*_oc,rad_(*J*_sun_) = 1.33 ± 0.02 eV, where the experimental error was mainly due to the uncertainty in the measurement of *T*. The effect of the dependence of the absorptivity on the layer thickness is within 10 meV (see Supplementary Note). Our all-optical value is in agreement with estimations of the radiative electrical voltage in HP solar cells, *V*_oc,rad_ = (1.32–1.34) ± 0.02 V, as independently assessed from electrical characteristics^20^.

The free energy *μ*_oc_ was estimated through eq. (2) by simply adding to the radiative limit *μ*_oc,rad_ the term *kT*ln(*EQY*_PL_) due to nonradiative recombinations. Experimental data show that *μ* is proportional to ln(*I*_ex_), with the slope coefficient giving the ideality factor. For 1 mW/cm^2^ < *I*_ex_ < 100 W/cm^2^, the slope was close to 3/2, independently of the method used to fabricate the MAPbI_3_ films, with an average ideality factor 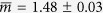.

In the light of the previous discussion on the *m*-rational values, experimental findings indicate that electrons and holes follow different power laws, and more specifically one carrier decays obeying a first order process (*α* = 1) while the other follows a second order process (*α* = 2). This asymmetry between electrons and holes is consistent with the presence of traps preferentially capturing a single carrier type. Figure 1 shows the recombination processes expected in a stand-alone, undoped HP layer, assuming empty traps^40^,^41^; fully occupied trap states would lead to similar conclusions, just swapping the roles of electrons and holes. The capture rate of electrons by intragap levels can be described as a bimolecular process, *R*_SRH,*e*_ ∝ *n*_*h,t*_*n*_*e*_, where *n*_*h,t*_ is the density of trapped holes. However, in the intrinsic semiconductor all traps are nearly empty, so *n*_*h,t*_ is almost equal to the density *N*_*t*_ of recombination centres. Consequently, the recombination process is monomolecular (*α*_*e*_ = 1). Similarly to the case of electrons, the hole capture rate is *R*_SRH,*h*_ ∝ *n*_*h*_*n*_*e,t*_, but in this case the densities of both trapped electrons *n*_*e,t*_ and free holes *n*_*h*_ increase with the excitation intensity and, additionally, *n*_*e,t*_~*n*_*h*_. Hole recombination is therefore an effective bimolecular process with *α*_*h*_ = 2; far from trap saturation, we obtain 
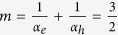
. We thus conclude that nonradiative recombinations in HPs can be described in the framework of the SRH model. In our samples, SRH recombination limits the available free energy to 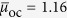 eV, with a loss at one sun excitation 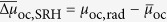 = 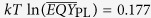 eV; lower losses could be achieved by reducing the trap density.

For *I*_ex_ > 100 W/cm^2^, *m* decreased to ~2/3, as expected for trimolecular annihilations via Auger decay channels. Over a wide range of excitation intensities, as in the *μ*_oc_-*J*_ex_ characteristics presented in this work, two (or more) elementary decay channels contribute to the recombination dynamics. Competition between them is such that the lowest-order electron-hole decays, identified by the lowest power index *α* and thus the highest *m* (nonradiative recombinations with *m* = 3/2 in perovskite films), dominates at low excitation intensities, while the highest order decay (nonradiative Auger recombinations with *m* = 2/3 in perovskite films) becomes the most important one at high excitation^14^,^27^,^42^,^43^. Similar behaviour is also observed in the *I*-*V* characteristics of a Si solar cell, where *m* decreases from 2 (SRH recombinations) to 1 for increasing voltage^28^. We rule out trap saturation because it would cause hole recombination to become monomolecular, as the population of trapped electrons becomes constant, and consequently *m* should increase from 3/2 to 2 (*α*_*e*_ = *α*_*h*_ = 1).

### Free energy and ideality factor in perovskite heterojunctions

According to the optical reciprocity relation in eq. (2), the larger *EQY*_PL_, the larger the resulting free energy. Therefore, a purely optical analysis of the *EQY*_PL_ ought to establish if additional interface recombination is setting stricter limits to *μ*_oc_ in single and double HP heterojunctions with respect to the bulk.

**Figure 3:**
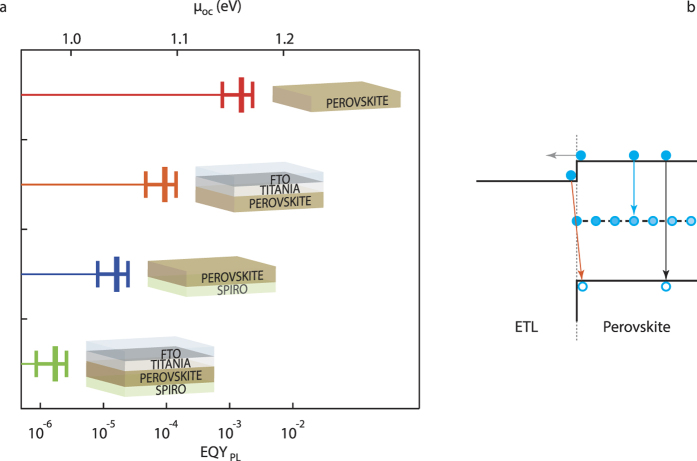
Comparison of the free electron-hole energy and external photoluminescence quantum yield in standalone perovskite layers and perovskite-based heterojunctions. (**a**) Top axis: experimental free energy *μ*_oc_ under 50 mW/cm^2^ CW laser excitation at 532 nm. Bottom axis: external photoluminescence quantum yield (*EQY*_*PL*_) under the same conditions. The rate of photons absorbed by the film matches that of an illumination level of one sun (AM 1.5G). Two types of single heterojunctions are reported: i-ETL (where the ETL is compact TiO_2_) and HTL-i (where the HTL is spiro-MeOTAD). Heterojunctions introduce non-radiative recombination channels, resulting in a lower free energy with respect to the single perovskite layer. (**b**) Schematic representation of electron-hole recombination processes in a heterojunction: bulk Shockley-Read-Hall decays (cyan arrow), radiative decays (black arrow) and interface decays (orange arrow); the sketch refers to the ETL side interface, a similar one would describe the HTL side.

Figure 3 shows the *EQY*_PL_ and corresponding *μ*_oc_ in these structures at an absorbed monochromatic photon density current corresponding to one sun. The standalone layer has the largest *EQY*_PL_, while significantly lower values are measured in the presence of interfaces, both in the i-ETL (perovskite-TiO_2_) and HTL-i (spiro-MeOTAD-perovskite) heterojunctions. Our interpretation of the evidence is that additional non-radiative recombination channels appear at both interfaces, providing faster non-radiative recombination than in the standalone layer. The full HTL-i-ETL structure, representing a contactless solar cell, shows an even lower *EQY*_PL_.

**Figure 4:**
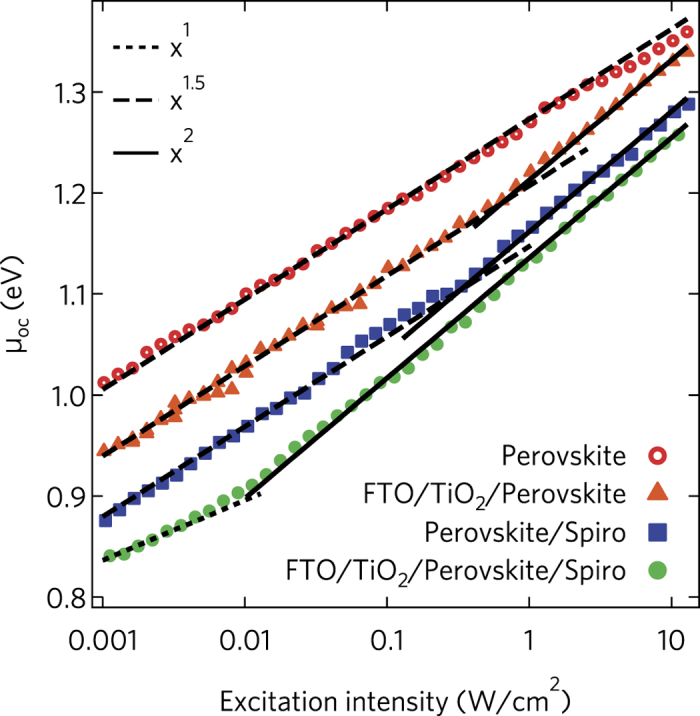
*μ*_oc_-*I*_ex_ characteristics and ideality factor of perovskite-based single and double heterojunctions. Markers represent the measured free energy *μ*_oc_ of electron-hole pairs in perovskite and perovskite-based heterojunctions as a function of the excitation intensity (*I*_ex_) delivered by a CW laser at 532 nm. Lines are provided as a guide to the eye to identify the slope of the data. The ideality factor deviates from the 1.5 value of the single hybrid perovskite layer, increasing to 2 when *I*_ex_ exceeds a threshold that is peculiar to each type of heterojunction.

The ideality factor in the heterojunctions was investigated by studying the *μ*-*I*_ex_ characteristics (Fig. 4), in the same way as for single HP layers (Fig. 2). Its value deviates from the 3/2 value measured in the intrinsic materials for *I*_ex_ between 0.01 and 100 suns. Furthermore, the *μ*_oc_-*I*_ex_ characteristics show that *m* increases with the excitation density, contrary to what expected from the competition between elementary recombination processes. *m* varies from ≈ 3/2 to ≈ 2 in single heterojunctions, and from ≈ 1 to ≈ 2 in double heterojunctions.

Possible interface recombination processes at the heterojunction interfaces are sketched in Fig. 3. In a steady-state experiment, electron (hole) transfer from the i-layer to the ETL (HTL) is compensated by electron (hole) back-transfer, preventing charge build-up at both sides of the interface. We attribute the lower *EQY*_PL_ observed in our perovskite heterojunctions with respect to the HP single layers to the activation of such interface decay channels^44^,^45^. Quenching of optical excitations at interfaces has even been exploited to measure the diffusion length in HPs^46^,^47^. Interface decay processes are not elementary decays and, as in the case of SRH decays, the ideality factor is expected to increase with the excitation intensity. Excitation-dependent band bending and modification of the energy level alignment close to the junctions, e.g. due to built-in electric fields, could drive non-linear phenomena for the carrier dynamics at the two interfaces. Anyway, the fact that at one sun *m* ≈ 2 suggests monomolecular recombinations for both electrons and holes (*α*_*e*_ ≈ *α*_*h*_ ≈ 1).

## Discussion

**Figure 5:**
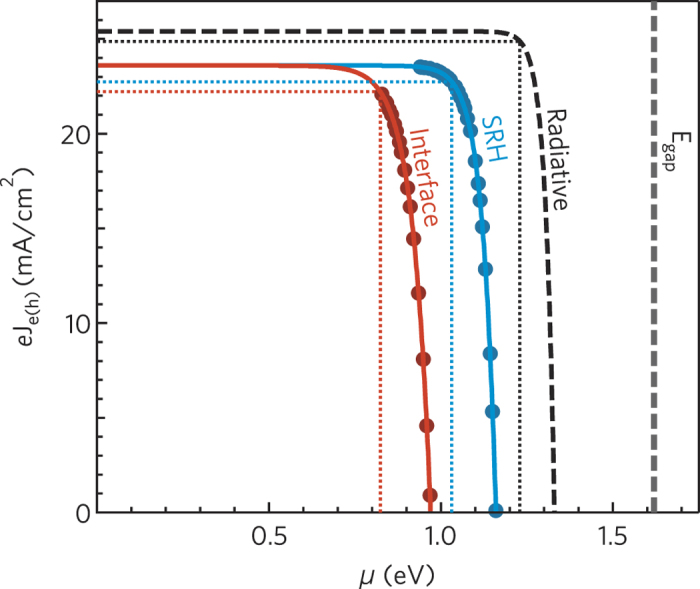
Electron (hole) charge current density as a function of the free energy. Solid lines represent the electron (hole) current *J*_*e(h*)_(*μ*) = *J*_sun_ − *J*_rec_, where *J*_sun_ is the excitation current at one sun (film thickness *d* = 250 nm), 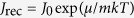 is the diode recombination current in which *m* and *J*_0_ are the experimental values of the ideality factor and saturation inverse current assessed from the *μ*-*I*_ex_ characteristics (Figs 2 and 4). Full circles stand for *J*_*e(h*)_(*μ*) estimated from the measured diode current *J*_rec_(*μ*_oc_) with the substitution *μ*_oc_ → *μ*. The dotted lines mark the point of maximum extraction of electrical power. Red curves and circles: electron (hole) current density in the double heterojunction; recombination is dominated by interface electron and hole annihilations. Cyan curves and circles: electron (hole) current density in the single hybrid perovskite layer; recombination is due to electron and hole Shockley-Read-Hall annihilations alone. Dashed line: *J*_*e(h*)_(*μ*) in the Shockley-Queissier limit.

Experimental results provide a detailed assessment of the conversion efficiency of the chemical energy *μ* into the electrical energy *eV*, under the assumption of no transport and carrier collection losses, but including all the effective recombination losses. To obtain an explicit estimate of the photoconversion efficiency, let us express the extracted electron (hole) current density from the continuity equation 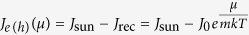, and the external electrical power as *J*_*e(h*)_*μ. J*_0_ can be experimentally estimated from the open circuit condition 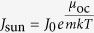. Figure 5 shows *eJ*_*e(h*)_(*μ*) and the condition for maximum chemical energy conversion, with efficiency 
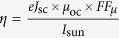
 (*FF*_*μ*_ being the filling factor of the *μ*-*J*_*e(h*)_ characteristics, and *eJ*_sc_ = *eJ*_sun_). The points in Fig. 5 are the experimental data *eJ*_ex_ = *eJ*_rec_(*μ*_oc_) from Figs 2 and 4 with the substitution 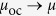
29. In the Shockley-Queissier limit, when the solar spectrum is perfectly absorbed down to the bandgap energy, with *μ*_rad_ = 1.33 eV and *m* = 1, the ultimate limit *η*_SQ_ = 30.5% is obtained (*eJ*_sc_ = 25.4 mA/cm^2^, *FF*_*μ*_ = 0.91). In the single HP layer, SRH limits the chemical energy to *μ*_oc_ = 1.16 eV and *m* = 1.5; furthermore, considering the actual absorption of a 250-nm thick HP film (as typical for solar cells reported in literature), the limiting photoconversion efficiency due to SRH recombination reduces to *η*_*SRH*_ = 23.4% (*eJ*_sc_ = 23.6 mA/cm^2^, *FF*_*μ*_ = 0.86). The strictest limit in the investigated double heterojunctions is set by interface recombinations to *μ* = 0.97 eV (*m* ≈ 2), resulting in *η* = 18.2% (*eJ*_sc_ = 23.6 mA/cm^2^, *FF*_*μ*_ = 0.80). Recently, solar cells have been reported based on HP materials with composite cations, including both organic molecules and the inorganic elements Rb and Cs, with very high external photoluminescence efficiency, up 3.6%^8^. Assuming no electrical losses, our analysis predicts for such cells a limit efficiency *η* = 26.2% (*d* = 400 nm, *eJ*_sc_ = 24.9 mA/cm^2^, *μ*_oc_ = 1.26, *FF*_*μ*_ = 0.83).

## Conclusions

The Shockley-Queissier model provides the reference, ultimate limit performance of single junction photovoltaic devices, assuming only radiative recombinations and neglecting all losses due to charge transport and extraction. We have developed an all-optical experimental method to assess the deterioration of photoconversion performances with respect to the Shockley-Queissier limit due to nonradiative recombinations. To this aim, the proposed approach allows measuring the upper limit to the open-circuit voltage of a solar cell set by nonradiative electron-hole decays in the intrinsic materials and assessing to what extent each interface in the device introduces additional recombination currents. We are able to identify the nature of decay processes, showing that recombinations in single perovskite layers can be described by the Shockley-Read-Hall model in the presence of defects that preferentially trap either electrons or holes. We estimate the ideality factor and the limit of the solar cell efficiency, without any current flowing through the device, therefore avoiding hysteresis, non-ideal contacts and charge transport losses.

## Methods

### Materials

All materials were purchased from Sigma-Aldrich or Alpha Aesar and used as received. Spiro-MeOTAD was purchased from Lumtec. CH_3_NH_3_I was synthesized according to a reported procedure^48^. CH_3_NH_3_ (27.86 ml, 40% in methanol, TCI) and hydroiodic acid (30 ml, 57 wt% in water, Aldrich) were mixed at 0 °C and stirred for 2 h. The precipitate was recovered byevaporation at 50 °C for 1 h. The product was washed with diethyl ether three times and finally dried at 60 °C in a vacuum oven for 24 h.

### Sample preparation

Glass substrates (Visiontech) and FTO-coated glass substrates (Solaronix) were cleaned by ultrasonication in a deionized water, 2-propanol and acetone. Substrates were treated to the TL1-washing procedure (washed in double distilled water (Milli-Q water), hydrogen-peroxide (H_2_O_2_) and ammonia (NH_3_) 5:1:1 v/v at 80 °C for 10 minutes), then rinsed in double distilled water prior next depositions. A 80 nm-thick TiO_2_ dense hole-blocking layer (ETL) was deposited on glass/FTO by spin coating a commercial titaniumdiisopropoxidebis(acetylacetonate) solution (75% in 2-propanol, Sigma-Aldrich) diluted in butanol (0,15 M) twice at 3,000 rpm for 60 sec and annealed at 125 °C. As last step a 0.3 M solution is spin-coated, and annealed at 520 °C. The prepared CH_3_NH_3_I and commercial PbI_2_ (99.99% ultradry, Alfa Aesar) were stirred in a mixture of γ-butyrolactone (GBL) and DMSO (2:1vol/vol; GBL, >99%; DMSO, 99.8%; Sigma-Aldrich) at 60 °C for 12 h, to prepare a solution 1 M. The perovskite precursor solution was coated onto either glass or TiO_2_/FTO substrate by a consecutive two-step spin-coating process at 1,000 and 4,000 r.p.m for 10 and 60 s, respectively with a dripping of dichloromethane at 10 s to the end. After spin-coating, the films were annealed on a hotplate at 100 °C for 10 min. After cooling to room temperature, either a PMMA solution (80 mg/1 mL chloroform) or the spiro-MeOTAD solution was spin-coated on the perovskite layer at 2,500 r.p.m. for 45 s. A spiro-MeOTAD solution was prepared by dissolving 90 mg of spiro-MeOTAD in 1 ml chlorobenzene (99.8%, Sigma Aldrich), to which were added 28.8 μl of 4-tert-butylpyridine (96%, Sigma-Aldrich), 17.5 *μ*l lithium bis (trifluoromethanesulfonyl)imide (LiTFSI) solution (520 mg LI-TSFI in 1 ml acetonitrile, 99.8%, Sigma-Aldrich). This fabrication process was carried out under controlled atmospheric conditions with a humidity of <1% and a temperature between 20 and 25 °C. From AFM measurements, we determined a perovksite film thickness of 160 nm.

### FTO/TiO_2_/perovskite/spiro-MeOTAD device fabrication

FTO-coated glass substrates (Solaronix) were cleaned by ultrasonication in deionized water, 2-propanol and acetone. A 80 nm-thick TiO_2_ dense hole-blocking layer (bl-TiO_2_) was then deposited on the substrates by spin coating at 3,000 rpm for 60 sec and annealed at 520 °C using a commercial titanium diisopropoxide bis(acetylacetonate) solution (75% in 2-propanol, Sigma-Aldrich) diluted in butanol (0,3 M). The prepared CH_3_NH_3_I and commercial PbI_2_ (99%, Alpha Aesar) for the 1 M CH_3_NH_3_PbI_3_ solution were stirred in a mixture of γ-butyrolactone (GBL) and DMSO (2:1 vol/vol; GBL, ≥99%; DMSO, 99.8%; Sigma-Aldrich) at 60 °C for 12 h. Before use, these precursor solutions were filtrated using a hydrophilic PTFE syringe filter (pore size of 22 μm). The filtrated perovskite precursor solution was coated onto bl-TiO_2_/FTO substrate by a consecutive two-step spin-coating process at 1,000 and 4,000 r.p.m for 10 and 60 s, respectively with a dipping of dicloromethan at 10 sec to the end. After spin coating, the films were annealed on a hotplate at 100 °C for 10 min. After cooling to room temperature, the spiro-MeOTAD solution was spin-coated on the perovskite layer at 2,500 r.p.m. for 45 s. A spiro-MeOTAD solution was prepared by dissolving 90 mg of spiro-MeOTAD in 1 ml chlorobenzene (99.8%, Sigma Aldrich), to which were added 28.8 μl of 4-tert-butylpyridine (96%, Sigma-Aldrich), 17.5 *μ*l lithium bis (trifluoromethanesulfonyl)imide (LiTFSI) solution (520 mg LI-TSFI in 1 ml acetonitrile, 99.8%, Sigma-Aldrich). This fabrication process was carried out under controlled atmospheric conditions with a humidity of <1% and a temperature between 20 and 25 °C. Finally, 80 nm gold was thermally evaporated on top of the device at a pressure of 5 × 10^−6^ mbar for 30 min to form the back contact. The active area of the complete device was 0.09 cm^−2^.

### Absolute photoluminescence quantum yield

*EQY*_PL_ was measured according to the method described by de Mello *et al*.^49^ using the beam from a diode-pumped Nd:YVO_4_ CW laser (Millennia V) at 532 nm as excitation. The irradiance of the beam was calculated as *E*_exc_ = *P/A*, where *P* is the optical power of the beam, measured by a bolometer, and *A* is beam area, measured using the knife-edge method. The samples were placed into an integrating sphere (Newport 819C-IS-5.3) and both the scattered laser light and the photoluminescence light were collected through a fibre-optic cable (Avantes FC-V200-1-SR) coupled to a grating spectrometer (Princeton Instruments Acton SpectraPro 2500i equipped with a 150 gr/mm, 600 nm blaze grating) and detected by a LN-cooled CCD camera. The laser beam was angled so that its reflection and/or the PL emission did not strike the output port directly. The spectral response of the detection system has been calibrated so that the number of counts is proportional to the number of photons collected. *EQY*_PL_ was calculated as:


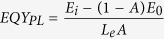


where *A* = (*L*_0_ − *L*_*i*_)/*L*_0_ and:

(a) *L*_*e*_ is the spectrally integrated intensity of the laser striking the inside of the empty integrating sphere;

(b) *L*_0_ is the spectrally integrated intensity of the laser striking the inside of the integrating sphere, with the sample inside the sphere but not under direct excitation;

(c) *E*_0_ is the spectrally integrated intensity of the photoluminescence from the sample under indirect excitation;

(d) *L*_*i*_ is the spectrally integrated intensity of the laser directly illuminating the sample;

(e) *E*_*i*_ is the spectrally integrated intensity of the photoluminescence as a result of direct excitation.

As required by the theoretical framework, the measurements were not corrected for self-absorption.

### Time-integrated photoluminescence (TIPL)

Samples were placed in a vacuum chamber and excited with a diode-pumped Nd:YVO_4_ CW laser (Millennia V) at 532 nm. The photoluminescence was dispersed with a grating spectrometer (Princeton Instruments Acton SpectraPro 2500i equipped with a 150 gr/mm, 600 nm blaze grating) and detected by a LN-cooled CCD camera.

## Additional Information

**How to cite this article**: Sarritzu, V. *et al*. Optical determination of Shockley-Read-Hall and interface recombination currents in hybrid perovskites. *Sci. Rep.* **7**, 44629; doi: 10.1038/srep44629 (2017).

**Publisher's note:** Springer Nature remains neutral with regard to jurisdictional claims in published maps and institutional affiliations.

## Supplementary Material

Supplementary Information

## References

[b1] Chapin D. M., Fuller C. S. & Pearson G. L. A New Silicon p-n Junction Photocell for Converting Solar Radiation into Electrical Power. J. Appl. Phys. 25, 676 (1954).

[b2] Best Research-Cell Efficiencies. NREL. *nrel.gov* Available at: http://www.nrel.gov/pv/assets/images/efficiency_chart.jpg (Accessed: 27 October 2016) (2016).

[b3] Tsai H. . High-efficiency two-dimensional Ruddlesden– Popper perovskite solar cells. Nature 536, 312–316 (2016). 27383783 10.1038/nature18306

[b4] Green M. A., Ho-Baillie A. & Snaith H. J. The emergence of perovskite solar cells. Nature Photon 8, 506–514 (2014).

[b5] Yang W. S. . High-performance photovoltaic perovskite layers fabricated through intramolecular exchange. Science 348, 1234–1237 (2015). 25999372 10.1126/science.aaa9272

[b6] Jung H. S. & Park N.-G. Perovskite Solar Cells: From Materials to Devices. Small 11, 10–25 (2015). 25358818 10.1002/smll.201402767

[b7] Sum T. C. & Mathews N. Advancements in perovskite solar cells: photophysics behind the photovoltaics. Energy Environ. Sci. 7, 2518–2534 (2014).

[b8] Saliba M. . Incorporation of rubidium cations into perovskite solar cells improves photovoltaic performance. Science 354, 206–209 (2016). 27708053 10.1126/science.aah5557

[b9] Bella F. . Improving efficiency and stability of perovskite solar cells with photocurable fluoropolymers. Science 354, 203–206 (2016). 27708051 10.1126/science.aah4046

[b10] Swarnkar A. . Quantum dot–induced phase stabilization of α-CsPbI_3_ perovskite for high-efficiency photovoltaics. Science 354, 92–95 (2016). 27846497 10.1126/science.aag2700

[b11] Shockley W. & Queisser H. Detailed Balance Limit of Efficiency of p‐n Junction Solar Cells. J. Appl. Phys. 32, 510–519 (1961).

[b12] McMeekin D. P. . A mixed-cation lead mixed-halide perovskite absorber for tandem solar cells. Science 351, 151–155 (2016). 26744401 10.1126/science.aad5845

[b13] Eperon G. E. . Perovskite-perovskite tandem photovoltaics with optimized band gaps. Science 354, 861–865 (2016). 27856902 10.1126/science.aaf9717

[b14] Manser J. S. & Kamat P. V. Band filling with free charge carriers in organometal halide perovskites. Nature Photon 8, 737–743 (2014).

[b15] Filippetti A. & Mattoni A. Hybrid perovskites for photovoltaics: Insights from first principles. Phys. Rev. B 89, 125203 (2014).

[b16] Yang Y. . Observation of a hot-phonon bottleneck in lead-iodide perovskites. Nat Photon 10, 53–59 (2015).

[b17] Grancini G. . Role of microstructure in the electron–hole interaction of hybrid lead halide perovskites. Nat Photon 9, 695–701 (2015). 10.1038/nphoton.2015.151 PMC4591469 26442125

[b18] Marchioro A., Teuscher J., Friedrich D. & Kunst M. Unravelling the mechanism of photoinduced charge transfer processes in lead iodide perovskite solar cells. Nature Photon 8, 250–255 (2014).

[b19] Saba M., Quochi F., Mura A. & Bongiovanni G. Excited State Properties of Hybrid Perovskites. Acc. Chem. Res 49, 166–173 (2015). 26696363 10.1021/acs.accounts.5b00445

[b20] Polman A., Knight M., Garnett E. C., Ehrler B. & Sinke W. C. Photovoltaic materials: Present efficiencies and future challenges. Science 352, 307 (2016). 10.1126/science.aad4424 27081076

[b21] Park N.-G., Grätzel M., Miyasaka T., Zhu K. & Emery K. Towards stable and commercially available perovskite solar cells. Nature Energy 1, 16152 (2016).

[b22] Ball J. M. & Petrozza A. Defects in perovskite-halides and their effects in solar cells. Nature Energy 1, 16149 (2016).

[b23] Shockley W. The Theory of p-n Junctions in Semiconductors and p-n Junction Transistors. Bell Labs Technical Journal 28, 435–489 (1949).

[b24] Green M. A. Radiative efficiency of state-of-the-art photovoltaic cells. Prog. Photovolt: Res. Appl. 20, 472–476 (2012).

[b25] Tvingstedt K. . Radiative efficiency of lead iodide based perovskite solar cells. Sci. Rep. 4, 6071 (2014). 25317958 10.1038/srep06071 PMC5377528

[b26] Tress W. . Predicting the open-circuit voltage of CH3NH3PbI3 perovskite solar cells using electroluminescence and photovoltaic quantum efficiency spectra: The role of radiative and non-radiative recombination. Adv. Energy Mater. 5, 1400812 (2015).

[b27] Colella S., Mazzeo M., Rizzo A., Gigli G. & Listorti A. The Bright Side of Perovskites. J Phys Chem Lett 7, 4322–4334 (2016). 27739681 10.1021/acs.jpclett.6b01799

[b28] Nelson J. The physics of solar cells. (Imperial College Press, 2003).

[b29] Würfel P. & Würfel U. Physics of solar cells: from basic principles to advanced concepts. (Wiley-VCH Verlag GmbH & Co. KGaA, 2009).

[b30] Agarwal S. . On the Uniqueness of Ideality Factor and Voltage Exponent of Perovskite-Based Solar Cells. J Phys Chem Lett 5, 4115–4121 (2014). 26278942 10.1021/jz5021636

[b31] Shi J. . Hole-conductor-free perovskite organic lead iodide heterojunction thin-film solar cells: High efficiency and junction property. Appl. Phys. Lett. 104, 063901 (2014).

[b32] Shockley W. & Read W. T. Jr. Statistics of the Recombinations of Holes and Electrons. Phys. Rev 87, 835–842 (1952).

[b33] Wetzelaer G. J. A. H. . Trap‐Assisted Non‐Radiative Recombination in Organic–Inorganic Perovskite Solar Cells. Adv. Mater. 27, 1837–1841 (2015). 25648930 10.1002/adma.201405372

[b34] Bi D. . Efficient luminescent solar cells based on tailored mixed-cation perovskites. Sci Adv 2, e1501170 (2016). 26767196 10.1126/sciadv.1501170 PMC4705040

[b35] Tvingstedt K. & Deibel C. Temperature Dependence of Ideality Factors in Organic Solar Cells and the Relation to Radiative Efficiency. Adv. Energy Mater. 6, 1502230 (2016).

[b36] Pockett A. . Characterization of Planar Lead Halide Perovskite Solar Cells by Impedance Spectroscopy, Open-Circuit Photovoltage Decay, and Intensity-Modulated Photovoltage/Photocurrent Spectroscopy. J. Phys. Chem. C 119, 3456–3465 (2015).

[b37] Würfel P. The chemical potential of radiation. J. Phys. C: Solid State Phys. 15, 3967–3985 (1982).

[b38] El-Hajje G. . Quantification of spatial inhomogeneity in perovskite solar cells by hyperspectral luminescence imaging. Energy Environ. Sci. 9, 2286–2294 (2016).

[b39] Rau U. Reciprocity relation between photovoltaic quantum efficiency and electroluminescent emission of solar cells. Phys. Rev. B 76, 085303 (2007).

[b40] Saba M. . Correlated electron–hole plasma in organometal perovskites. Nat Comms 5, 5049 (2014). 10.1038/ncomms6049 25266869

[b41] Delugas P., Filippetti A. & Mattoni A. Methylammonium fragmentation in amines as source of localized trap levels and the healing role of Cl in hybrid lead-iodide perovskites. Phys. Rev. B 92, 045301 (2015).

[b42] Cadelano M. . Can Trihalide Lead Perovskites Support Continuous Wave Lasing? Advanced Optical Materials 3, 1557–1564 (2015).

[b43] Wehrenfennig C., Eperon G. E., Johnston M. B., Snaith H. J. & Herz L. M. High Charge Carrier Mobilities and Lifetimes in Organolead Trihalide Perovskites. Adv. Mater. 26, 1584–1589 (2014). 24757716 10.1002/adma.201305172 PMC4722848

[b44] Zhou H. . Interface engineering of highly efficient perovskite solar cells. Science 345, 542–546 (2014). 25082698 10.1126/science.1254050

[b45] Correa-Baena J.-P. . Highly efficient planar perovskite solar cells through band alignment engineering. Energy Environ. Sci. 8, 2928–2934 (2015).

[b46] Xing G. . Long-Range Balanced Electron- and Hole-Transport Lengths in Organic-Inorganic Ch3NH3PbI3. Science 342, 344–347 (2013). 24136965 10.1126/science.1243167

[b47] Stranks S. D. . Electron-hole diffusion lengths exceeding 1 micrometer in an organometal trihalide perovskite absorber. Science 342, 341–344 (2013). 24136964 10.1126/science.1243982

[b48] Lee M. M., Teuscher J., Miyasaka T., Murakami T. N. & Snaith H. J. Efficient Hybrid Solar Cells Based on Meso-Superstructured Organometal Halide Perovskites. Science 338, 643–647 (2012). 23042296 10.1126/science.1228604

[b49] De Mello J. C., Wittmann H. F. & Friend R. H. An Improved Experimental Determination of External Photoluminescence Quantum Efficiency, Adv. Mater. 9, 230 (1997).

