# Supplementary material for: Optical determination of Shockley-Read-Hall and interface recombination currents in hybrid perovskites
Source: Sci Rep. 2017 Mar 20;7:44629. doi: 10.1038/srep44629 (PMC5357960; doi:10.1038/srep44629)
Supplement: Supplementary Information [file srep44629-s1.pdf]

Supplementary information for the Article

Optical determination of Shockley-Read-Hall and interface recombination currents in hybrid perovskites

Valerio Sarritzu,[a] Nicola Sestu,[a] Daniela Marongiu,[a] Xueqing Chang,[a] Sofia Masi,[b] Aurora Rizzo,[b] Silvia Colella,[b,c] Francesco Quochi,[a] Michele Saba*,[a] Andrea Mura,[a] Giovanni Bongiovanni*.[a]

[a]Dipartimento di Fisica, Università degli Studi di Cagliari, I-09042 Monserrato, Italy.

[b]Istituto di Nanotecnologia CNR-Nanotec, Polo di Nanotecnologia c/o Campus Ecotekne, via Monteroni, 73100 Lecce, Italy,

[c]Dipartimento di Matematica e Fisica "E. De Giorgi", Università del Salento, Via Arnesano snc, 73100 Lecce, Italy

Supplementary Note 1 | Radiative electron-hole free energy $\mu_{\mathrm{oc,rad}}$

The Fermi level of the unexcited film and the trap level intersect in the middle of the intrinsic layer, where recombination centres are thereby half-filled ($\alpha_e = \alpha_h = 1, m = 2$). In this region, the annihilation rate $R_{\mathrm{SRH},e} = R_{\mathrm{SRH},h}$ is higher than in proximity of the transport layers, where it is almost suppressed. Hence, the different occupation of the intragap states is at the origin of the different ideality factors in HTL-i-ETL structures ($m = 2$) and in single i-layers ($m = 3/2$).

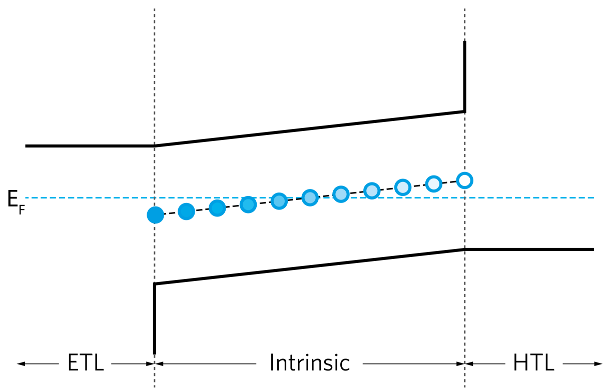

Supplementary Figure 2 | Energetics of an ETL-i-HTL double heterojunction in the dark. Equilibrium conditions of the solar cell in the dark impose that the concentration of trapped electrons varies across the intrinsic layer: trap levels at the centre of the i-semiconductor traps are half-filled.

Supplementary Note 2 | Radiative electron-hole free energy $\mu_{\mathrm{oc,rad}}$

The radiative free energy per electron-hole pair is defined as

$$\mu_{\mathrm{oc,rad}} = kT \ln\left(\frac{J_{\mathrm{ex}}}{J_{0,\mathrm{rad}}}\right) \quad (1)$$

To find an analytical expression of $\mu_{\mathrm{oc,rad}}$, the radiative emission $J_{0,\mathrm{rad}}$ of the semiconductor layer in thermal equilibrium with the environment at temperature T is reformulated as follows:

$$J_{0,rad} = \frac{\Omega}{4\pi^3\hbar^3{c_0}^2}\int_0^{\infty} a(E)E^2 e^{-\frac{E}{kT}}\mathrm{d}E =$$

$$\bar{a}_T \frac{\Omega}{4\pi^3\hbar^3{c_0}^2}\int_{E_\mathrm{L}}^{\infty} E^2 e^{-\frac{E}{kT}}\mathrm{d}E = \bar{a}_T \frac{\Omega}{4\pi^3\hbar^3{c_0}^2} kT\, {E_\mathrm{L}}^2\, e^{-\frac{E_L}{kT}}\left[2\left(\frac{kT}{E_\mathrm{L}}\right)^2 + 2\frac{kT}{E_\mathrm{L}} + 1\right] \quad (2)$$

where E is equal to the photon energy $\hbar\omega$. The equivalent absorptivity $\bar{a}_T$ is defined as:

$$\bar{a}_T = \frac{\int_0^{\infty} a(E)E^2 e^{-\frac{E}{kT}}\mathrm{d}E}{\int_{E_\mathrm{L}}^{\infty} E^2 e^{-\frac{E}{kT}}\mathrm{d}E} \quad (3)$$

while

$$\int_{E_\mathrm{L}}^{\infty} E^2 e^{-\frac{E}{kT}}\mathrm{d}E = kT\, {E_\mathrm{L}}^2\, e^{-\frac{E_\mathrm{L}}{kT}}\left[2\left(\frac{kT}{E_\mathrm{L}}\right)^2 + 2\frac{kT}{E_\mathrm{L}} + 1\right] \quad (4)$$

and E_L is a suitable low energy cut-off parameter to evaluate the integral. Similarly, the solar excitation current reads:

$$J_\mathrm{ex} = \frac{\Omega_\mathrm{sun}}{4\pi^3\hbar^3{c_0}^2}\int_0^{\infty} a(E)E^2 e^{-\frac{E}{kT_\mathrm{sun}}}\mathrm{d}E$$

$$= \bar{a}_{sun} \frac{\Omega_{sun}}{4\pi^3\hbar^3{c_0}^2} k\, T_{sun}{E_L}^2\, e^{-\frac{E_L}{kT_{sun}}}\left[2\left(\frac{kT_{sun}}{E_L}\right)^2 + 2\frac{kT_{sun}}{E_L} + 1\right] (5)$$

where:

$$\bar{a}_{sun} = \frac{\int_0^{\infty} a(E)E^2 e^{-\frac{E}{kT_\mathrm{sun}}}\mathrm{d}E}{\int_{E_\mathrm{L}}^{\infty} E^2 e^{-\frac{E}{kT_\mathrm{sun}}}\mathrm{d}E} (6).$$

A natural choice for E_L is the optical gap $E_\mathrm{gap} = 1.602$ eV, namely the lowest excitonic transition energy. As discussed in recent reports,[1,2] after resonant excitation bound excitons quickly ionize to form free electron-hole carriers in 3D halide perovskites. According to these definitions, $\mu_\mathrm{oc,rad}$ reads:

$$\mu_\mathrm{oc,rad} = kT\ln\left(\frac{J_\mathrm{ex}}{J_{0,rad}}\right) = E_\mathrm{gap}\frac{T_\mathrm{sun}-T}{T_\mathrm{sun}} + kT\ln(\frac{T_\mathrm{sun}}{T}) + kT\ln[f\left(T, T_\mathrm{sun}, E_\mathrm{gap}\right)\frac{\Omega_\mathrm{sun}\bar{a}_\mathrm{sun}}{\Omega\, \bar{a}_T}] \; (7)$$

where

$$f(T, T_{\text{sun}}, E_{\text{PL}}) = \frac{2\left(\frac{kT_{\text{sun}}}{E_{\text{gap}}}\right)^2 + 2\frac{kT_{\text{sun}}}{E_{\text{gap}}} + 1}{2\left(\frac{kT}{E_{\text{gap}}}\right)^2 + 2\frac{kT}{E_{\text{gap}}} + 1} \quad (8).$$

T_{sun} was chosen in such a way that the photon current density emitted into the solid angle $\Omega_{\text{sun}} = 6.8 \text{ x } 10^{-5}$ sr from a blackbody at T_{sun} was equal to the tabulated solar photon current density at AM1.5G for $\hbar\omega > E_{\text{gap}}$. According to Lambert's law[3], the effective solid angle in equation (2) is $\Omega = \pi$. We finally stress that $\mu_{\text{oc,rad}}$ given in equation (7) is nothing but a different way to formulate the radiative free energy at open circuit, as no approximation has been made to derive it from equation (1). In fact, the spectra integration involved in the definition of $J_{0,rad}$ and J_{ex} is simply contained in the definition of $\bar{a}_T$ and $\bar{a}_{\text{sun.}}$ The utility of equation (7) stems from two considerations: i) in the SQ limit $\frac{\bar{a}_{\text{sun}}}{\bar{a}_T} = 1$, and thus $\mu_{\text{oc,rad}}$ has an useful analytical expression that allows an easy estimate of the radiative free energy, $\mu_{\text{oc,rad}} = 1.33$ eV ; ii) as a matter of fact, the effects of the finite film thickness and of the not sharp edge absorption are small, leading to a shift of the free energy $\Delta\mu_{\text{oc,rad}} \approx 10$ meV, which means that the analytical SQ value is an excellent approximation in perovskite films.

For the calculation of $\mu_{\text{oc,rad}}$ from equation (1), we operated as follows. For simplicity, the absorptivity of all structures was approximated as the absorptivity of a single slab of thickness d, with a back reflector (reflectivity $r_b = 1$) simulating the back metal contact of a solar cell:

$$a(E) = [1 - r(E)]\left[1 - e^{-2\alpha(E)d}\right]\left[1 - r(E)e^{-2\alpha(E)d}\right] \quad (9)$$

where $\alpha(E)$ is the perovskite absorption coefficient and $r(E)$ the reflectivity of the front surface. Supplementary Figure 1 shows the experimental absorptivity, the experimental photoluminescence spectrum (PL_{exp}) and the spectrum of the radiative emission spectrum ($J_{0,rad}$), as predicted by the Kirchhoff's law of radiation:

$$J_{0,rad}(E) \propto a(E)E^2 e^{-\frac{E}{kT}} \quad (10)$$

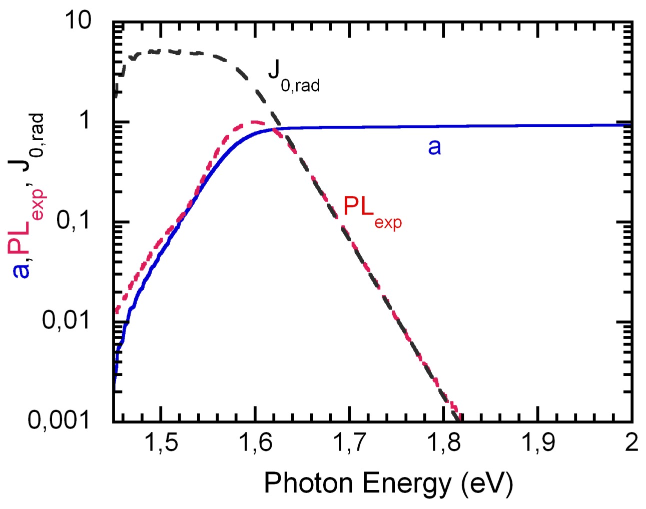

Supplementary Figure 1 | Experimental (PL_{exp}) and theoretical ($J_{0,rad}$) photoluminescence spectra. The latter was estimated via equation (S1), and the experimental absorptivity a.

Due to light scattering, the measurement of the absorption coefficient $\alpha(E)$ was overestimated below the gap. This slowly decaying tail did not compensate for the raising Boltzmann function, resulting in a distorted spectral shape. To overcome this experimental uncertainty, we assumed that $J_0(E)$ has the same spectral shape of $PL_{\mathrm{exp}}(E)$, that is $J_{0,\mathrm{rad}}(E) = PL_{\mathrm{exp}}(E)\,\frac{J_{0,\mathrm{rad}}(\bar{E})}{PL_{\mathrm{exp}}(\bar{E})}$, where $\bar{E} = E_{\mathrm{gap}} + 50$ meV. J_{ex} was simply calculated as $\int_0^{\infty} a(E)J_{\mathrm{sun}}(E)dE$, where $J_{\mathrm{sun}}(E)$ is the solar photon current density at AM1.5G. With this approximations, we calculated $\mu_{\mathrm{oc,rad}}$ according to equation (1). We found that the estimated free energy was equal to the value found in the SQ limit, within an uncertainty of approximately 10 meV.

References

1. D'Innocenzo, V. *et al.* Excitons versus free charges in organo-lead tri-halide perovskites. *Nat. Commun.* **5,** 3586 (2014).
2. Saba, M., Quochi, F., Mura, A. & Bongiovanni, G. Excited State Properties of Hybrid

Perovskites. *Acc. Chem. Res.* **49,** 166–173 (2016).

3. Würfel P., Würfel U. *Physics of Solar Cells: From Basic Principles to Advanced Concepts*. (Wiley-VCH, Weinheim, 2016).
